# Support for the reproductive ground plan hypothesis of social evolution and major QTL for ovary traits of Africanized worker honey bees (*Apis mellifera *L.)

**DOI:** 10.1186/1471-2148-11-95

**Published:** 2011-04-13

**Authors:** Allie M Graham, Michael D Munday, Osman Kaftanoglu, Robert E Page, Gro V Amdam, Olav Rueppell

**Affiliations:** 1 Department of Biology, University of North Carolina at Greensboro, 1000 Spring Garden Street, 312 Eberhart Building, Greensboro, NC, 27403, USA; 2 Current address: Duke University, Department of Biology, Box 90338 Durham, NC 27708, USA; 3 School of Life Sciences, Arizona State University, PO Box 874501, Tempe, AZ 85287-4501, USA; 4 Department of Chemistry, Biotechnology and Food Science, Norwegian University of Life Sciences, 1432 Aas, Norway

**Keywords:** Reproductive groundplan hypothesis, Social evolution, Complex trait locus mapping, Pollen hoarding syndrome, Worker reproduction, Asymmetry

## Abstract

**Background:**

The reproductive ground plan hypothesis of social evolution suggests that reproductive controls of a solitary ancestor have been co-opted during social evolution, facilitating the division of labor among social insect workers. Despite substantial empirical support, the generality of this hypothesis is not universally accepted. Thus, we investigated the prediction of particular genes with pleiotropic effects on ovarian traits and social behavior in worker honey bees as a stringent test of the reproductive ground plan hypothesis. We complemented these tests with a comprehensive genome scan for additional quantitative trait loci (QTL) to gain a better understanding of the genetic architecture of the ovary size of honey bee workers, a morphological trait that is significant for understanding social insect caste evolution and general insect biology.

**Results:**

Back-crossing hybrid European x Africanized honey bee queens to the Africanized parent colony generated two study populations with extraordinarily large worker ovaries. Despite the transgressive ovary phenotypes, several previously mapped QTL for social foraging behavior demonstrated ovary size effects, confirming the prediction of pleiotropic genetic effects on reproductive traits and social behavior. One major QTL for ovary size was detected in each backcross, along with several smaller effects and two QTL for ovary asymmetry. One of the main ovary size QTL coincided with a major QTL for ovary activation, explaining 3/4 of the phenotypic variance, although no simple positive correlation between ovary size and activation was observed.

**Conclusions:**

Our results provide strong support for the reproductive ground plan hypothesis of evolution in study populations that are independent of the genetic stocks that originally led to the formulation of this hypothesis. As predicted, worker ovary size is genetically linked to multiple correlated traits of the complex division of labor in worker honey bees, known as the pollen hoarding syndrome. The genetic architecture of worker ovary size presumably consists of a combination of trait-specific loci and general regulators that affect the whole behavioral syndrome and may even play a role in caste determination. Several promising candidate genes in the QTL intervals await further study to clarify their potential role in social insect evolution and the regulation of insect fertility in general.

## Background

The Western honey bee (*Apis mellifera *L.) is an important pollinator and scientific model, particularly for studying social evolution and complex behavior. The reproductive ground plan hypothesis (RGPH) of social evolution has been suggested to explain the evolution of several aspects of honey bee biology, particularly behavioral specialization in the helper caste of workers [1-3]. Based on the ovarian ground plan hypothesis [4,5], the RGPH proposes that control modules of the ancestral gonotrophic cycle of a hypothetical solitary ancestor have been co-opted by social evolution, influencing honey bee worker behavior and life history. Thus, hormones and genes are predicted to pleiotropically influence worker reproductive traits and nest provisioning behavior (foraging).

The RGPH has been supported by an increasing number of studies [1,2,6-9], accumulating evidence for associations between reproductive traits of honey bee workers and their social behavior and life history. These studies have used ovary size, a convenient reproductive trait because it varies greatly among individuals and can be easily quantified by dissection as the number of parallel ovary filaments, the ovarioles. Ovary size co-varies with the age at which workers transition from in-hive tasks to foraging in different populations of *A. mellifera *[1,10]. After foraging initiation, many foragers specialize on either pollen or nectar collection and this specialization is also related to ovary size in *A. mellifera *[1,6] and the Eastern honey bee, *Apis cerana *[7]. Furthermore, ovary size correlates with other aspects of the pollen hoarding syndrome in worker honey bees [8], such as sucrose responsiveness [1,9].

In addition to these phenotypic correlations, the RGPH predicts mechanistic links between reproductive and social behavioral regulation, which have been demonstrated by the study of candidate genes, such as vitellogenin [11-13] and genes associated with insulin-like signaling [6,14]. However, the most stringent test of the RGPH consists of the demonstration of common genetic variation for reproductive traits and social behavior that segregates in contemporary bee populations. Genetic variation is implicated in enhancing the division of labor in honey bee colonies [15] but not expected to result from selection on worker ovary size *per se *[16].

Artificially selected high and low pollen hoarding strains of honey bees [17] were instrumental for the initial formulation of the RGPH and detection of phenotypic associations between worker reproductive traits and social behavior [1-3]. These selected strains have also been used to establish the genetic co-segregation between ovary size and foraging specialization [6]. However, the generality of the relation between reproductive traits and foraging specialization in honey bees has been questioned because this relation was not found in a study of another selection line (the "anarchistic line", characterized by the unusual occurrence of worker reproduction in the presence of a queen) [10]. Therefore, more general tests of co-segregating genetic variation for social behavior and reproductive traits are warranted to evaluate the RGPH.

One such independent test system in the Western honey bee (*Apis mellifera*) is provided by the Africanized population in South and North America. It has originated through hybridization of an introduced *A. mellifera scutellata *ancestor from Africa with honey bees of different subspecies of European descent, characterized by phenotypic and genomic displacement of the European by the African ancestor [18,19]. Compared to the European honey bees (EHB) in America, Africanized bees (AHB) are more responsive to sucrose, transition earlier to foraging tasks, and forage more for pollen [20]. The ovariole number of AHB workers is also higher than that of their EHB counterparts [21,22], but see [23]. Even though independent, this system is similar to the selected pollen hoarding strains at the phenotypic level: the AHB differ from the EHB in the same way that high pollen hoarding strain bees differ from low pollen hoarding strain bees. The AHB/EHB system has been used previously to confirm quantitative trait loci (QTL) for foraging specialization that had been initially discovered in the selected pollen hoarding strains [24]. Thus, crosses between colonies selected from the AHB and EHB populations provide an independent test system of the prediction of genetic co-segregation of social behavior and ovary size.

In total, four QTL for pollen hoarding behavior and foraging specialization have been mapped in the honey bee genome [24-26]. These QTL (*pln1 *- *pln4*) were repeatedly confirmed [24,26] and found to pleiotropically affect other aspects of the pollen hoarding syndrome [27,28]. They are located on chromosome 1 and 13 and are significantly enriched for genes involved in insulin-like signaling [29]. Additional QTL for the age of first foraging (*aff1 *- *aff4*) and for sucrose responsiveness (*per1*) were identified [27,28] but only three *aff *QTL could subsequently be located to specific genome locations on chromosome 4, 5, and 11 [30]. Testing for genetic effects of the seven located *pln *and *aff *QTL on worker ovary size provide specific tests of a central prediction of the RGPH.

The genetic architecture of ovary size has been studied in detail in *Drosophila*, suggesting major, interacting QTL and environmental effects [31-33], but little is known about other insect species. In social insects, ovary size is particularly important in the context of differential fertility between the female worker and queen castes [34]. Honey bees have evolved a large caste difference: queen ovaries typically contain >100 ovarioles and workers <10 [21]. Probably due to the evolution of this large phenotypic plasticity, intra-specific variability within the worker caste is also high, with significant population differences [21] and strong variability within populations [35] and even among sibling crosses [22,23,36].

In a series of crosses between AHB and EHB, worker ovary size showed a transgressive inheritance pattern [22]: The parental AHB had more ovarioles per ovary than the parental EHB source, with hybrids intermediate. EHB backcrosses resulted in workers with ovariole numbers that were similar to the parental EHB, but AHB backcrosses showed much larger ovary sizes than their parents. In two of these crosses, workers had ovaries with as many as 39 and 74 ovarioles [22], suggesting that segregating genetic variation in workers can lead to phenotypic differences in the same order of magnitude as caste differences. Thus, the genetic basis of worker ovary size variation may be based on the same mechanisms that control caste differences [22].

A preliminary analysis of these two crosses identified one strongly supported QTL on chromosome 11 and several weaker ones as the potential genetic basis for these large worker ovary sizes via selective, pooled QTL mapping [22]. However, this fast and cost-effective approach has previously generated results that could not be subsequently verified by individual genotyping [30,36]. The present study examines an extended population of workers with a transgressive ovary size from the two crosses analyzed previously [22]. Our first objective is to specifically test for pleiotropic effects of the *pln *and *aff *QTL on ovary size, as predicted by the RGPH. Secondly, we extend our preliminary analysis [22] towards a comprehensive understanding of the genetic architecture of the transgressive worker ovary trait by individual genotyping and analyzing different aspects of worker ovary size and worker ovary activation under queenless conditions.

## Results

### Ovary phenotypes

**Figure 1:**
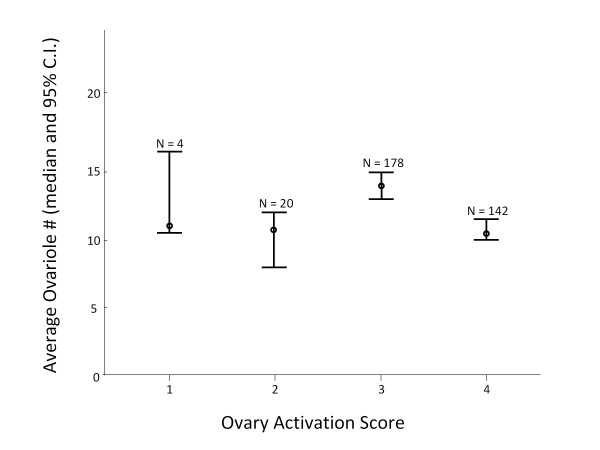
**The relationship between ovary size and degree of ovary activation under queenless conditions in Africanized backcross workers that are characterized by large ovaries**. Almost all workers have developed their ovaries to some extent. The workers with the largest ovaries only had an activation score of three in contrast to some workers with smaller ovaries that contained fully developed eggs.

In the first backcross, ABC3, the minimum ovary size ranged from 2 to 31 ovarioles with a median of 11 (quartiles: 7.25 - 15.0), maximum ovary size ranged from 3 to 31 ovarioles with a median of 13 (9.25 - 17.0) and average ovary size from 2.5 to 31 ovarioles with a median of 11.75 (8.5 - 16.0). The differences between the larger and the smaller ovary ranged from 0 - 10 ovarioles with a median of 2.0 (1.0 - 3.0), translating into relative differences from 0 to 0.50 with a median of 0.067 (0.037 - 0.133), and ratios from 0.33 - 1.0 with a median of 0.875 (0.766 - 0.929). In the parallel backcross, ABC5, the minimum ovary size ranged from 4 to 28 ovarioles with a median of 11 (8.0 - 14.0), maximum ovary size ranged from 4 to 30 ovarioles with a median of 14 (11.0 - 17.0), and average ovary size from 4 to 29 ovarioles with a median of 12 (10.0 - 15.375). The differences between the larger and the smaller ovary ranged from 0 - 15 ovarioles with a median of 2. 0 (1.0 - 4.0), translating into relative differences from 0 to 0.47 with a median of 0.09 (0.04 - 0.17), and ratios from 0.36 - 1.0 with a median of 0.83 (0.71 - 0.92). Ovary activation scores ranged from 1 - 4 with a median of 3 (3 - 4). Despite asymmetric ovaries in 85.2% (ABC3) and 86.9% (ABC5), ovary size between the two body sides was highly correlated (Table 1). The overall negative correlation between ovary size and activation score in ABC5 was caused by a non-linear relation between these two variables, combined with unequal representation of the different ovary activation classes in our sample (Figure 1). Individuals with an activation score of three had significantly larger ovaries than individuals with activation scores of two (Kruskal Wallis' H = 94.1, post-hoc p < 0.001) or four (H = 77.3, p < 0.001) but the latter effect outweighed the former due to unequal sample sizes (Figure 1). Except for the relationship between minimum ovary size and the difference or ratio between the two ovary sides, ABC3 and ABC5 show very similar relations between the different variables (Table 1).

**Table 1:** Correlations* among ovary variables in ABC3 (n = 88, above diagonal) and ABC5 (n = 344, below diagonal)

	Minimum ovary size	Maximum ovary size	Average ovary size	Difference in ovary size	Relative difference	Ratio of ovary size
Minimum ovary size	---	R_S _= 0.95, p < 0.001	R_S _= 0.98, p < 0.001	R_S _= 0.03, p = 0.763	R_p _= -0.41, p < 0.001	R_p _= 0.41, p < 0.001

Maximum ovary size	R_S _= 0.80, p < 0.001	---	R_S _= 0.99, p < 0.001	R_S _= 0.31, p = 0.003	R_S _= -0.14, p = 0.190	R_S _= 0.14, p = 0.190

Average ovary size	R_S _= 0.94, p < 0.001	R_S _= 0.95, p < 0.001	---	R_S _= 0.19, p = 0.080	R_S _= -0.26, p = 0.013	R_S _= 0.26, p = 0.013

Difference in ovary	R_S _= -0.20, p < 0.001	R_S _= 0.35, p < 0.001	R_S _= 0.09, p = 0.104	---	R_S _= 0.85, p < 0.001	R_S _= -0.85, p < 0.001

Relative difference	R_S _= -0.50, p < 0.001	R_S _= 0.04, p = 0.465	R_S _= -0.23, p < 0.001	R_S _= 0.94, p < 0.001	---	R_S _= -1.0, p < 0.001

Ratio of ovary size	R_S _= 0.50, p < 0.001	R_p _= -0.04, p = 0.465	R_S _= 0.23, p < 0.001	R_S _= -0.94, p < 0.001	R_S _= -1.0, p < 0.001	---

Ovary activation	R_S _= -0.23, p < 0.001	R_S _= -0.25, p < 0.001	R_S _= -0.26, p < 0.001	R_S _= -0.03, p = 0.630	R_S _= 0.04, p = 0.520	R_S _= -0.04, p = 0.520

* Spearman's correlations (R_S_) were used. For explanation of variables, see "Methods" in main text.

### QTL Analyses

**Figure 2:**
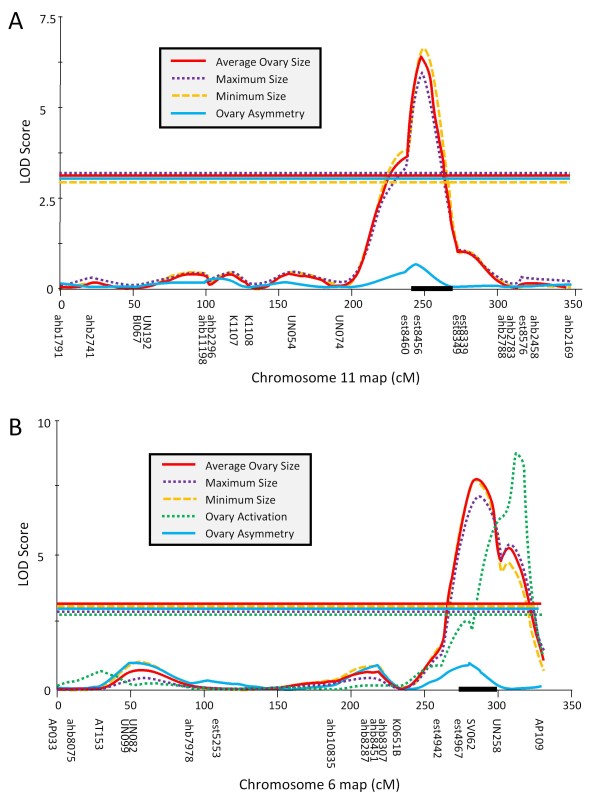
**In both parallel backcrosses one major QTL for transgressive ovary size in worker honey bees was identified**. However, these major QTL were on different chromosomes in the ABC3 backcross (a) and the ABC5 backcross (b).

**Figure 3:**
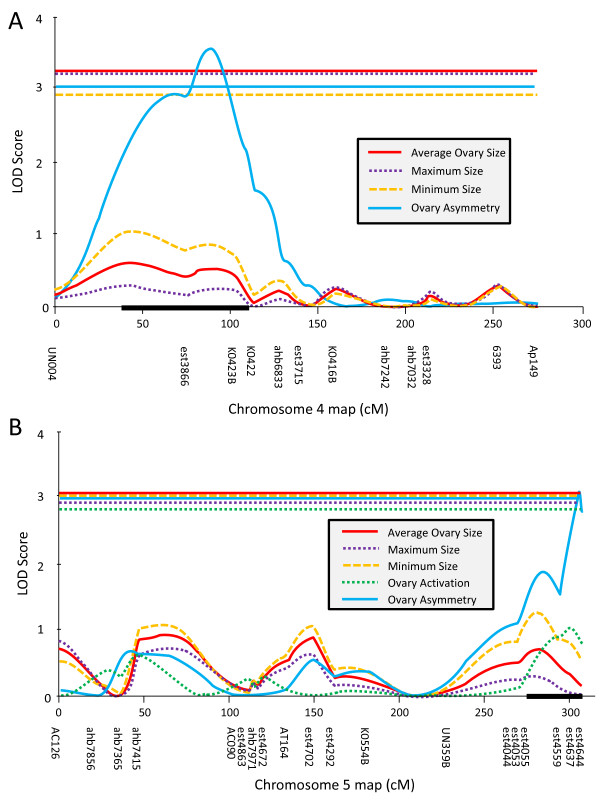
**In both parallel backcrosses one significant QTL for ovary asymmetry in worker honey bees was identified**. The ABC3 QTL was located on the 4^th ^chromosome (a) and the ABC5 QTL was located on the 5^th ^chromosome (b), indicating no overlap between the backcrosses.

The final ABC3 map contained 137 SNP and 98 microsatellite markers with an average inter-marker interval of 20.2 cM and 94.4% of the mapable genome within 20 cM of at least one genetic marker. Most of the top single markers (Table 2) were represented in the QTL indicated by interval mapping (Table 3): One major QTL for ovary size was mapped to chromosome 11 (Figure 2a). LOD support of this QTL well above significance and it explained about 1/3 of the phenotypic variance in the mapping population for all ovary size traits but had only a subtle effect on ovary asymmetry measures (Table 3). Two additional, suggestive QTL for ovary size were found on chromosome 5, approximately 5 cM proximal from marker AT137 and on chromosome 10 between markers K1055 and K1064 (Table 3). One significant QTL was found for ovary asymmetry on chromosome 4 between marker est3866 and K0423B (Figure 3a) with an effect that was independent of ovary size (Table 3).

**Table 2:** The most significant single markers in the study with an uncorrected significance of <0.01.

	Average ovary size	Minimum ovary size	Maximum ovary size	Ovary asymmetry	Ovary development
ABC3	est8456 (C.11, p < 0.0001)	est8456 (C.11, p < 0.0001)	est8456 (C.11, p < 0.0001)	est3866 (C.4, p < 0.001)	N/A
	est8460 (C.11, p < 0.0001)	est8460 (C.11, p < 0.0001)	est8460 (C.11, p < 0.0001)	K0423B (C.4, p < 0.005)	N/A
	AT137 (C.5, p < 0.005)	AT137 (C.5, p < 0.005)	ahb2105 (C.10, p < 0.01)		N/A
	ahb2105 (C.10, p < 0.01)	ahb2105 (C.10, p < 0.01)	A040 (C.1, p < 0.01)		N/A
	est1833 (C.2, p < 0.01)		AT137 (C.5, p < 0.01)		N/A

ABC5	est4967 (C.6, p < 0.0001)	est4967 (C.6, p < 0.0001)	est4967 (C.6, p < 0.0001)	est4637, C.5, p < 0.0005)	est4967 (C.6, p < 0.0001)
	SV062 (C.6, p < 0.0001)	SV062 (C.6, p < 0.0001)	SV062 (C.6, p < 0.0001)	est4644, C.5, p < 0.001)	UN258 (C.6, p < 0.0005)
	est10110 (C.13, p < 0.0005)	est10110 (C.13, p < 0.001)	est10110 (C.13, p < 0.0001)	ahb10918 (C.1, p < 0.005)	K1551 (C.15, p < 0.005)
	UN258 (C.6, p < 0.001)	UN258 (C.6, p < 0.005)	UN258 (C.6, p < 0.0005)	est6265 (C.8, p < 0.01)	est1929 (C.2, p < 0.01)
	est10066 (C.13, p < 0.005)	est8339 (C.11, p < 0.005)	est10066 (C.13, p < 0.005)	ahb12014 (C.8, p < 0.01)	

*C. = Chromosome

**Table 3:** Statistics for the QTL detected by interval mapping in cross ABC3 (MQM scores in brackets).

QTL	Average ovary size	Minimum ovary size	Maximum ovary size	Ovary asymmetry*
Chromos. 11 (Figure 2a)	LOD = 6.5 (8.3), 35.4% Var. expl.	LOD = 6.7 (6.7), 38.8% Var. expl.	LOD = 5.9 (7.0), 29.7% Var. expl.	LOD = 0.0 - 2.0 (0.0 - 2.6), 0.0 - 9.7% Var. expl.

Chromos. 5	LOD = 2.0 (1.6), 16.4% Var. expl.	LOD = 2.3 (1.4), 18.2% Var. expl.	LOD = 1.7 (1.1), 14.4% Var. expl.	LOD = 0.5 - 1.1 (0.1 - 0.2), 5.1 - 13.5% Var. expl.

Chromos. 10	LOD = 1.9 (3.1), 16.0% Var. expl.	LOD = 1.6 (2.5), 14.0% Var. expl.	LOD = 2.0 (3.0), 16.8% Var. expl.	LOD = 0.0 - 0.6 (0.0 - 0.9), 0.2 - 3.6% Var. expl.

Chromos. 4 (Figure 3a)	LOD = 0.5 (0.3), 3.5% Var. expl.	LOD = 0.9 (1.6), 5.6% Var. expl.	LOD = 0.3 (0.5), 1.8% Var. expl.	LOD = 2.6 - 4.0 (2.6 - 4.2), 18.0 - 28.9% Var. expl.

Chromos. 2	LOD = 1.4 (2.3), 6.8% Var. expl.	LOD = 1.3 (1.8), 6.8% Var. expl.	LOD = 1.3 (2.2), 6.6% Var. expl.	LOD = 0.0 - 0.6 (0.1 - 0.2), 1.1 - 4.0% Var. expl.

* Values describe the range of 3 different asymmetry measures.

MQM analysis increased the LOD score of this asymmetry QTL and the statistical support for an effect of the QTL on chromosome 11 on ovary asymmetry (Table 3). MQM also increased the LOD score of the ovary size effects of the QTL on chromosome 11 and the suggestive QTL on chromosome 10 (Table 3) but decreased LOD scores for the suggestive QTL on chromosome 5. For average ovary size, MQM also indicated another suggestive QTL on chromosome 2 near marker est1833.

None of the pairwise epistasis tests among the significant and suggestive QTL was significant after Bonferroni correction. The empirically determined, genome-wide LOD significance thresholds were 2.9 for minimum ovary size, and 3.2 for maximum and average ovary size. Thresholds for ovary asymmetry ranged from 2.9 to 3.1, depending on the specific measurement.

**Figure 4:**
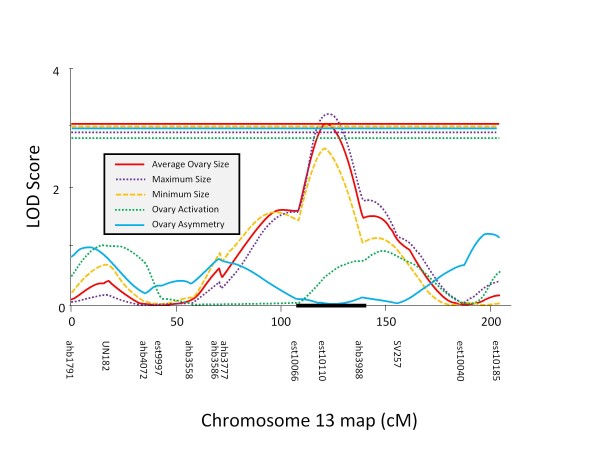
**An additional significant QTL for ovary size was identified in the ABC5 backcross population**. This QTL is identical to the behavioral *pln1 *QTL, demonstrating pleiotropy as predicted by the reproductive ground plan hypothesis of social evolution in honey bees.

The final map of ABC5 contained 149 SNP and 82 microsatellite markers with an average inter - marker interval of 18.7 cM and 91.7% of the mapable genome within 20 cM of at least one genetic marker. Again, most top-scoring single markers (Table 2) were located in the QTL identified by interval mapping (Table 4): One major QTL for all ovary size traits was found on chromosome 6 between markers est4967 and UN258. The region had no effect on asymmetry measure but strongly influenced the ovary activation score (Table 4), although the LOD trace diverged from the other traits (Figure 2b). Another significant QTL for ovary size but not ovary asymmetry or activation (Table 4) was located on chromosome 13, centered on marker est10110 (Figure 4). The two significant ovary size QTL did not interact (F_(1,182) _= 0.5, p = 0.465).

**Table 4:** Statistics for the QTL detected by interval mapping in cross ABC5.

QTL	Average ovary size	Minimum ovary size	Maximum ovary size	Ovary activation score	Ovary asymmetry*
Chromos. 6 (Figure 2b)	LOD = 7.8, 14.5% Var. expl.	LOD = 7.8, 15.0% Var. expl.	LOD = 7.2, 14.1% Var. expl.	LOD = 8.7, 74.0% Var. expl.	LOD = 0.1 - 1.1, 0.2 - 2.7% Var. expl.

Chromos. 13 (Figure 4)	LOD = 3.1, 4.8% Var. expl.	LOD = 2.6, 4.2% Var. expl.	LOD = 3.2, 5.5% Var. expl.	LOD = 0.8, 2.2% Var. expl.	LOD = 0.1 - 0.8, 0.2 - 2.4% Var. expl.

Chromos. 5 (Figure 3b)	LOD = 0.4, 0.6% Var. expl.	LOD = 0.9, 1.4% Var. expl.	LOD = 0.1, 0.2% Var. expl.	LOD = 1.0, 3.1% Var. expl.	LOD = 3.0 - 3.1, 6.9 - 7.2% Var. expl.

* Values describe the range of 3 different asymmetry measures.

A significant QTL for ovary asymmetry but not ovary size or activation (Table 4) was found on chromosome 5 between markers est4576 and est4637 (Figure 3b). No further, suggestive QTL for any traits were detected. The empirically determined, genome-wide significant thresholds were 3.0 for minimum and average ovary size, 2.9 for maximum ovary size, and 2.8 for ovary activation score. Significance thresholds ranged from 2.9 for "relative difference" and 3.0 for "absolute difference" to 3.3 for "ratio", excluding chromosome 3 which produced a discrete group of unreasonably large LOD scores of >10 for the asymmetry variables. MQM was precluded by the selective genotyping strategy in ABC5.

In ABC3, only the genetic marker near the *aff4 *QTL showed a significant effect on ovary size. In ABC5, markers near three of the previously determined behavioral QTL showed a significant effect on ovary size (*pln1*, *pln2*, *aff2*) and the effect of *pln3 *was suggestive (Table 5). There were two overlaps between the three new ovary size QTL and the seven previously mapped, behavioral QTL when a wide confidence interval of the indeterminate [30]*aff *QTL on chromosome 11 was assumed. This or more overlap has a probability of 0.017 to occur by chance in two crosses. Assuming a narrow confidence interval for the *aff *QTL, only one overlap between the ovary and behavioral QTL was detected (p = 0.332).

**Table 5:** Genetic effects of previously identified behavioral QTL on average ovary size

Backcross	QTL	Marker	Mann-Whitney test	Effect size (# of ovarioles)
ABC3	*AFF4*	At164	U_(N = 87) _= 1182.5 p = 0.035	2.8

ABC5	*PLN1*	est10110	U_(N = 186) _= 3008.5, p < 0.001	2.8
	
	*PLN2*	AT110	U_(N = 94) _= 1423.0, p = 0.016	2.7
	
	*PLN3*	est788	U_(N = 189) _= 3702.5, p = 0.052	1.4
	
	*AFF2*	ahb2647	U_(N = 186) _= 5211.5, p = 0.015	1.9

### Candidate genes

**Figure 5:**
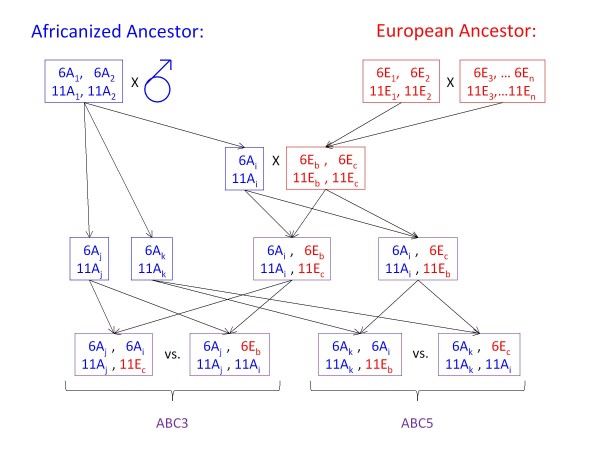
**Possible genetic model of the inheritance of the observed transgressive ovary sizes, focusing on the two major QTL on chromosome 6 and chromosome 11 in the two backcrosses ABC3 and ABC5**. Possibly different alleles are indicated by subscripts. The crossing scheme does not allow for different alleles from the Africanized ancestor to segregate at one locus. Thus, the differences between ABC3 and ABC5 must be caused by dominance or epistasis effects.

For each significant QTL, the 1.5 LOD support interval [29] depicted as a black bar on the x-axis of Figures 2, 3, 4 and 5, was searched for positional candidates in the NCBI database. The full list of the current (Amel4.0) annotation of the positional candidates is available as online supplement. For the major QTL on chromosome 11 (Figure 2a) 54 gene models were predicted, 3 of which were hypothetical loci. The functionally most interesting genes in this list were the orthologs of *quail *(LOC410324) and *cabut *(LOC410326), but it was also noteworthy that the *notch *ortholog (LOC410351) was located just outside the QTL confidence interval. In the second major ovary size QTL region on chromosome 6 (Figure 2b), 47 positional candidates were present. Two loci were hypothetical and functional candidates included the *seven-up *receptor ortholog (LOC408872), the transcription factor *anormal oocyte *ortholog (LOC551371), and the putative steroid hydrogenase LOC725258. The third significant ovary size QTL on chromosome 13 (Figure 4) contained 34 gene models with significant similarity to known genes and 1 hypothetical locus. It partially overlapped with *pln1 *and thus contained some of the same functional candidate genes (orthologs of *bazooka*: LOC726759 and *midway*: LOC552377) but also orthologs of *Ajuba *(LOC408431), *abrupt *(LOC726491), and *toucan *(LOC726183). The ovary asymmetry QTL on chromosome 5 contained 147 positional candidate gene models. Twelve models were hypothetical loci and some functionally interesting genes were the *Putative Achaete Scute Target 1 *ortholog (LOC413012), the *Nedd8 *ortholog LOC552822, the ortholog of *CTP:phosphocholine cytidylyltransferase 1 *(LOC412303), and the *pebble *ortholog LOC413063. In the support interval of the ovary asymmetry QTL on chromosome 4, 83 total gene models included 12 hypothetical loci. Among the remainder, noteworthy genes of known functions were the *vitellogenin *gene, the *coro *ortholog LOC409316, and the *MIG-2*-*like *ortholog LOC552138.

## Discussion

As predicted by the RGPH of social evolution in honey bees, worker ovary size showed genetic overlap with QTL of two key aspects of worker social behavior, the age of first foraging [30] and foraging specialization [29]. Thus, our study provides a strong, independent confirmation of the RGPH by demonstrating genetic effects of behavioral QTL on ovary size in two crosses that are unrelated to the selected high and low pollen hoarding strains. The analyzed crosses differ dramatically in their ovarian phenotypes from these selected strains [36] and most worker honey bees in general [22]. Despite the phenotypic distinctiveness of the studied bees, the RGPH prediction of phenotypic [22] and genotypic linkage between the ovary and social behavior has been supported. Together with previous QTL mapping studies [24-26,28], our results indicate that pronounced, co-segregating genetic variation for worker ovary size and social behavior is maintained in contemporary honey bees. The magnitude of the QTL effects on ovary size suggests either a link to the evolution of caste differences [22] or a significant role of this variation in colony function [37].

With direct, pleiotropic effects in four of fourteen QTL tests in this system, the detected genetic overlap in ABC3 and ABC5 is stronger than in crosses between the high and low pollen hoarding strains, which showed two effects in eight tests [6]. Such tests of pleiotropy in quantitative traits are conservative in general because the absence of an effect could be due to a lack of segregating variation in the specific cross studied or due to genetic background and environmental effects that may affect the penetrance of the QTL effect. Accordingly, previous studies of genetic overlap between components of the pollen hoarding syndrome have found pleiotropic effects only in 3/12 tests of *pln *QTL effects on sucrose responsiveness [27] and in 1/8 tests of *pln *QTL effects on the age of first foraging [28]. Our study reports the highest proportion of genetic overlap, focusing on foraging behavior and the ovary. Accounting for the marginal *pln3 *effect further strengthens this argument, increasing the proportion of positive tests to 5/14. In addition, the effect size of *pln1*, and presumably also *aff2*, on ovary size were sufficiently pronounced to be detected at the genome wide significance threshold for mapping novel QTL. The overlap between *aff2 *and the ovary size QTL on chromosome 11 was only detected with after widening the confidence interval for *aff2*. However, this is justified because the initial interval was arbitrarily narrow, spanning only 11 cM [30]. In addition, evidence for widening the confidence interval of the ovary size QTL exists when the evidence from ABC3 and ABC5 is combined (see Figure eight in [22]).

This study has also discovered novel QTL for ovarian traits of honey bee workers in regions that are not known for any behavioral effects. Future studies will need to address whether these QTL also show behavioral effects. The partial genetic overlap among different aspects of the pollen hoarding syndrome, which has been reported previously [27,28,36] corresponds best to a genetic network with a combination of central, pleiotropic regulators and downstream, specific effectors. This interpretation is compatible with our molecular understanding of worker ovary size determination during larval development, which is dependent on general regulators that influence all aspects of caste differentiation, such as juvenile hormone [34], but progresses through very specific mechanisms, such as actin-spectrin interactions and apoptosis [38,39].

The intervals for the three significant ovary size QTL contain 135 positional candidate genes in total, with several candidates that have either a general or a specific putative molecular role that make them plausible functional candidates. Orthologs of at least 16 transcription factors and 7 members of major signaling pathways are present as candidates of potentially general function, and orthologs of 2 apoptosis-related and 2 actin-associated genes may represent functional candidates that are involved in the specific downstream processes that determine worker ovary size [39]. In addition, there may be numerous unannotated transcripts, particularly regulatory RNA with a potential role in ovary development that we are not able to discuss. Our top candidates for the QTL on chromosome 11 are the ortholgs of *quail *and *cabut*: *Quail *is a villin-like protein that is active in various life history stages in the *Drosophila *ovary and interacts with actin [40], which makes it a potential specific effector on worker ovary size [39]. *Cabut *is a transcriptional activator that is responsive to TOR [41] and ecdysone [42] signaling, involved in the JNK cascade [43] and autophagic cell death [44]. Moreover, *cabut *shows developmental expression differences between the high and low pollen hoarding strains [45]. In addition, the chromosome 11 QTL interval contains the NFAT transcription factor related LOC408354, which is differentially expressed in female larval development [46] and LOC408367, the ortholog of a NADH dehydrogenase and three unidentified transcripts that all differ in expression between worker and queen developing ovaries (Klaus Hartfelder, pers. commun.).

We consider the ortholog of the *seven-up *receptor as the top functional candidate for the second major QTL (on chromosome 6) because *seven-up *is a nuclear receptor that can inhibit ecdysteroid signaling [47], controls cell proliferation [48], and interacts with *Krüppel *[49], a gene that has been implied in reproductive regulation in workers of honey bees and bumble bees [50]. We consider the transcription factor *abnormal oocyte *(LOC551371) a second top candidate because preliminary data show an exceptionally high dN/dS substitution ratio in this gene when *A. dorsata*, whose workers have very high ovariole numbers [51], is compared to *A. mellifera *(Dawid Adnew, Ryan Kuster, Olav Rueppell, unpublished data). The top candidates for the QTL on chromosome 13 are the orthologs of *Ajuba*, a negative regulator of the *Hippo *pathway that mediates tissue size by controlling cell proliferation and apoptosis [52], *midway*, a diacylglycerol acyltransferase gene that has been linked to actin reorganization and apoptosis in the *Drosophila *ovary [53] and *bazooka*, a regulator of IIS signaling [29] that shows differential expression between the high and low pollen hoarding strains [45]. In addition, a putative AMP-binding, fatty acid Co-A ligase gene (LOC726040) and the fumarylacetoacetase gene (LOC552210) in this interval show differential expression [46]. Functional candidates for ovary asymmetry are even harder to prioritize because they include genes that could influence ovary size (e.g. *vitellogenin*) and genes dealing with stress resistance, such as a *cytochrome P450 monooxygenase *(Cyp314a1) and *Glutathione S transferase S1 *(LOC411045).

Regardless of the molecular mechanism, our results show that there are at least three major and several minor QTL segregating in the two parallel crosses that we have analyzed. The phenotypic effects range from large, significant QTL to the pleiotropic effects of some of the behavioral QTL that could only be detected by specific tests. The most pronounced QTL was identified in ABC3 on chromosome 11, with an allelic substitution effect at the nearest marker (est8456) of six ovarioles for average ovary size, explaining over 1/3 of the phenotypic variance in this cross. This major effect could explain the bimodality of ovary size in ABC3 workers in certain environments [22]. Caste development in general is a threshold process and it is possible that this QTL affects a specific threshold for ovary development, causing a major increase in ovary size that is modulated by minor loci and environmental effects, including indirect genetic effects [54]. A minor effect of this region on ovary size was detected in ABC5 and this QTL was also identified by our preliminary QTL mapping based on selective, pooled genotyping of the same crosses [22]. In addition, the other ovary size QTL that had been indicated by the preliminary study in ABC3 [22] was detected by the individual analysis as a suggestive QTL on chromosome 10. In contrast, none of the QTL that were initially identified only from ABC5 (on chromosomes 2, 4, and 8) could be reconfirmed. This difference in concordance could be explained by the higher overlap of individuals used in the preliminary and this study in ABC3 than in ABC5. However, the lack of overlap between the two studies in ABC5 could also be due to differences in statistical power or methodological problems that can compromise pooled genotyping results [36].

The second major QTL was detected in ABC5 on chromosome 6 with a similar allelic substitution effect on ovary size (5.8 ovarioles) but no genotypic effect of this region was detected in the parallel cross ABC3. The consistent differences between the two crosses can only be explained by non-additive genetic effects in these backcrosses (Figure 5). The two queen mothers of ABC3 and ABC5 share any allele from their AHB father but only 50% of the alleles from their EHB mother. Both backcrosses were sired by brother AHB drones that share any specific allele with 50% probability. However, these paternal alleles are not segregating in the backcrosses. Since the increase in ovary size is derived from the AHB ancestor [22] and the segregating AHB alleles are identical between ABC3 and ABC5, the effect of these segregating alleles must depend on the identity of another allele, either at the same or at a different locus (Figure 5) to explain the differences between the parallel ABC3 and ABC5 crosses. Such dominance or epistatic effects are ubiquitous in the genetic architecture of the pollen hoarding syndrome [26-28,36]. Our results suggest that these effects may have also played a part in the evolution of caste divergence, shielding certain segregating alleles for reproductive potential from selection.

Previously it had been suggested that at least two interacting loci with a recessive allele for large ovary size were responsible for the phenotype of ABC3 and ABC5, as well as several other parallel backcrosses that did not show a high ovariole number [22]. The current results indicate that the genetic basis is more complex with three significant QTL, two suggestive QTL, and several loci of minor influence, such as the *pln *and *aff *QTL. The most likely explanation is that ABC3 workers are fixed for a "large" ovary allele combination at several loci (e.g. on chromosome six: *6A*_*j*_/*6A*_*i *_= *6Aj*/*6E *_*b *_in Figure 5) that elevates the average ovary size and allows segregating variation at the QTL on chromosome 11 (Figure 5: *11A*_*i*_/*11A*_*j *_≠ *11E*_*c*_/*11A*_*j*_) to manifest itself. Conversely, ABC5 workers carry alternative allele combinations that make only a small difference in ovary size at the chromosome 11 QTL (Figure 5: *11A*_*i*_/*11A*_*k *_≈ *11E*_*b*_/*11A*_*k*_) but a large difference at the chromosome 6 QTL (Figure 5: *6A*_*i*_/*6A*_*k *_≠ 6*E*_*c*_/6*A*_*k*_). Additionally, several segregating alleles of minor effects may contribute to the elevated ovariole numbers in ABC5. Alternatively, differential parental imprinting [55] could explain the different phenotypes and QTL effects in the parallel crosses.

Following [36], we analyzed ovary size as a composite variable consisting of a smaller and a larger side. Although the correlation between the two sides was high in both backcrosses, the two variables were affected slightly differently by the QTL. Minimum ovary size showed a stronger association with genotype at most, but not all QTL. It may be that minimum size is less prone to environmental influences than maximum size. The two ovary size variables were also combined into different measures of asymmetry to assess the intra-individual plasticity of ovary size. The main conclusions did not differ significantly among the three specific measures. One QTL for ovary asymmetry without an effect on ovary size was identified in each cross. This is in contrast to our results in two different crosses [36] and demonstrates genetic elements in these regions that influence either fluctuating or directional asymmetry of the ovary size in honey bee workers. Based on our measurements, we cannot distinguish between directional and fluctuating asymmetry [56], but directional asymmetry seems more likely for the following reasons: Evidence for directional asymmetry in worker ovaries exists [57], major QTL are more likely to be present for directional than for fluctuating asymmetry [58], and no other striking asymmetries were noticeable in bees with asymmetric ovaries.

In ABC5, we took the opportunity to analyze the degree of worker ovarian activation under queenless conditions and found one strong QTL on chromosome 6, explaining approximately 3/4 of the phenotypic variation. This extremely high value suggests monogenetic inheritance but it is likely an overestimate due to the categorical nature of the variable "ovary activation". Although the LOD traces are different between this ovary activation QTL (most significant single marker: UN258) and the ovary size QTL (most significant single marker: SV062) in this genome region (Figure 1b), the two QTL overlap extensively and may be due to the same molecular variant. The region does not overlap with any of the minor QTL for ovary activation reported in the anarchistic strains that can activate their ovaries in the presence of the queen [59] and thus we conclude that the degree of ovary activation with and without a queen are not necessarily related.

In contrast to a previous study [16], the ovary size and the degree of ovary activation in the 14-day old workers of the ABC5 sample were not linearly correlated. Instead, workers with the largest ovaries often showed only an activation score of 3, while workers with slightly smaller ovaries more often had maximally developed ovaries. This effect could be due to a combination of the very large ovaries observed in this cross and the competition for food when almost all workers start to develop their ovaries under queenless conditions. Almost all workers in ABC5 had developed their ovaries to some extent. Workers with very large ovaries may not have had sufficient nutrients in the absence of supporting workers [60] to simultaneously activate their many ovarioles as effectively as workers with slightly smaller ovarioles [22]. These data suggest that there may be an optimal worker ovary size for individual worker reproduction when a colony becomes hopelessly queenless. In contrast, the maintenance of the observed extensive genetic variation for ovary size is likely to be due to selection on co-opted, new functions of the reproductive control modules.

## Conclusion

The presented data provides further support for the RGPH of social evolution by demonstrating that several behavioral QTL also affect ovary size in worker honey bees. In addition, significant novel QTL were detected for worker ovary size and asymmetry, as well as the degree of ovary activation under queenless conditions and a genetic model to explain the extreme phenotypes was proposed. Evidence for non-additive effects exists, although pair-wise epistasis among the novel QTL could be excluded in both crosses. Some functionally interesting candidate genes exist in these QTL that need to be studied further. The exceptional phenotypic variation of the two investigated crosses makes our results relevant for the mechanistic understanding of honey bee caste divergence and allowed us to test the RGPH in an independent study system, exploiting a novel phenotypic space.

## Methods

### Mapping populations

This study investigated the same two crosses that were analyzed by Linksvayer et al. (2009). In brief, 12 European honey bee (EHB) and 12 Africanized honey bee (AHB) colonies were screened for ovary size. The phenotypic extremes were crossed to produce hybrid queens by artificial insemination with sperm from a single drone. These were then backcrossed by artificial insemination to both parental colonies. The two analyzed crosses in this study were both backcrosses to the AHB parent that showed the highest mean and variance of worker ovary size when reared in unrelated host colonies [22]. All 88 workers with complete phenotypic information and high-quality DNA of the 94 collected workers from the most extreme backcross (ABC3) were used as mapping population. The second backcross (ABC5) was selectively genotyped, focusing on the 190 individuals with the most extreme overall ovary size of the 344 workers with complete data. ABC3 workers were dissected directly after emergence. In contrast, ABC5 workers were kept in two unrelated host hives for two weeks before collection. The host hives had been made queen- and brood-less 10 days prior to the introduction of newly emerged ABC5 workers. This treatment leads to ovary activation in honey bee workers [61], allowing the simultaneous analysis of ovary size and activation.

### Phenotyping

The abdomen was separated from head and thorax, pinned ventral side up into a dissection dish, and opened with two lateral cuts. After removal of the sternites, both ovaries were carefully dissected out, placed on a microscope slide, and viewed with a dark field compound microscope in order to count the number of ovarioles present in each ovary. The combination of the two counts provided measures of minimum, maximum, and average ovary size for each individual. In addition, three measures of ovary asymmetry were calculated: the difference between the sides, the relative difference (difference divided by the sum), and the ratio between the smaller to the larger value. Furthermore, ovary activation was scored using a 5-point scale [62]: 0 = undeveloped (resting ovarioles); 1 = oogenesis starting (presence of cells swelling at top of ovariole and starting to descend); 2 = slight development (eggs distinguishable from trophocytes); 3 = moderate development (egg volume exceeds that of the follicle); 4 = highly developed (eggs are fully elongated). Since most variables showed significant departures from normality, non-parametric statistics were used where possible, employing the PASW 18.0 software package (SPSS Inc. Chicago, Il).

### Genotyping

DNA was extracted from the head and thorax, using a CTAB-phenol/chloroform protocol [25]. The concentration of the DNA was quantified with a Nanodrop^® ^spectrophotometer and all samples diluted to 100 ng/ml. These stocks were then diluted to template solutions of 10 ng/μl in low TE (0.3 mM EDTA). Based on the results of pooled genotyping of select individuals of both backcrosses with a panel of 1536 SNPs [22], 280 of these SNPs were selected for individual genotyping. This genotyping was performed by MALDI-TOF mass spectrometry (Sequenom, CA) with automated genotype calling [63], according to Sequenom's internal company standards. The SNPs were genotyped in all 88 individuals of ABC3 and the 190 phenotypic extremes in ABC5. Monomorphic loci and loci with <50% successful base calling rates were omitted from the analysis.

For any remaining gaps in genome coverage that were >30 cM, additional microsatellite markers located in these genome regions were genotyped. These markers were adapted from a published genome map of >2000 microsatellites [64] or designed from the published honey bee genome sequence [65]. Microsatellite regions were PCR-amplified using a tailed primer approach [66] to allow fluorescent detection of the PCR products on a LiCor (Lincoln, Nebraska) Automated Sequencer (DNA Analyzer 4300). For all loci a single touchdown PCR protocol was used, decreasing the annealing temperature from 68°C to 48°C [67]. PCR reactions were performed in 10 μl, containing 10 ng of template DNA, 200 μM dNTPs, 120 nM forward primer, 360 nM reverse primer, 50 nM of IRD-labeled M13 primer, 2 mM MgCl_2_, standard PCR buffer, and 0.2u of Taq polymerase. PCR products were separated by electrophoresis on denaturing, 6% polyacrylamide gels (length: 25 cm, thickness 0.25 mm) at 1000V for 2-3 hours. Microsatellite markers were first amplified in a set of eight random individuals to determine amplicon size by comparison to appropriate size standards (LiCor, Lincoln, Nebraska) and screen for polymorphism and amplification. Subsequently, suitable markers were amplified in all 88 (ABC3) or 96 (ABC5) individuals and PCR products of different size and IRD label were multiplexed for genotype determination by gel electrophoresis.

### Analyses

SNP and microsatellite data were combined and initially assembled into linkage groups according to their predicted physical genome locations. Using Mapmaker 3.0 [68] this marker order was then verified by maximum likelihood analysis and linkage distances between markers estimated, using the Kosambi map function. Markers that led to a significant (>5% and >5 cM) map extension or deviation from the high-density reference map [64] were re-genotyped and/or rescored to exclude flawed data. In cases when assembly problems persisted, the responsible marker was removed from the data set or it was placed in a different genome region as determined by linkage analysis. Between-marker intervals that differed in length by more than 50% between our maps and the high-density reference map [64] were reduced to the smaller interval length as a conservative basis for the subsequent QTL mapping because large gaps might produce artifactual QTL [30].

QTL mapping was performed with the MapQTL 4.0 software package [69]. Single marker analyses and interval mapping was used in all cases, but multiple QTL model (MQM) mapping was only used in ABC3 because the selective genotyping of ABC5 does not allow MQM analysis [69]. For all analyses, a LOD ≥ 2 was considered suggestive and included as co-factors in MQM mapping and a LOD ≥ 3 was considered significant [25]. In addition, empirical significance thresholds were determined for each trait and cross separately by genome-wide permutation tests [70]. Pair-wise epistasis between all identified significant and suggestive QTL in both backcrosses was tested by assessing the interaction terms of two-factorial ANOVAs using the nearest genetic marker as factors. Significance thresholds were Bonferroni-corrected to account for the multiple testing. Higher-order interactions could not be evaluated in a meaningful way due to limited sample size. The 1.5 LOD support intervals of each QTL were directly determined from the interval mapping LOD functions to define the 97% confidence interval for each QTL location [29].

The genotypic effect on average ovary size was evaluated for one closely linked SNP or microsatellite marker near each *pln *and *aff *QTL to test for the predicted pleiotropy of these QTL. As an additional, more conservative test of overlap between segregating genetic variation for worker ovary size and previously mapped behavioral QTL, the exact probability of overlap between the *pln *and *aff *QTL and the detected QTL for ovary size was calculated. The meeting probability of the *i*^*th *^ovary QTL with any of the behavioral QTL was computed as , where *w*_*i *_is the fraction of the 97% CI of the *i*^*th *^ovary QTL to the overall physical size of the honey bee genome (236 Mb; [65]) and *w*_*T *_is the ratio of the combined length of the 97% CI of the behavioral QTL to overall genome size. These probabilities were multiplied to calculate the probability of multiple QTL overlaps and summed to account for independent possibilities of a given amount of overlap.

All candidate genes in the 97% confidence intervals of the QTL were determined from the official GLEAN set [65] to search for functional candidate genes. All genes and putative gene entries were evaluated based on differential gene expression [46] and known functions of their homologs, as listed in the NCBI database http://www.ncbi.nlm.nih.gov/gene and FLYBASE http://flybase.bio.indiana.edu/.

## Authors' contributions

REP, GVA, and OR conceived and designed the experiments. The crosses were generated and evaluated by OK and genotyping was performed by AMG, MDM, and OR. AMG and OR performed the analyses and drafted the manuscript. AMG, GVA, and OR wrote the final version of the manuscript but all authors contributed to the writing, read and approved the final manuscript.
